# Carbon-Ion Radiation Therapy for Unresectable Solitary Fibrous Tumor of the Intermediate Category: A Nationwide Multi-institutional Observational Study by the Japan Carbon-Ion Radiation Oncology Study Group

**DOI:** 10.1016/j.adro.2026.102143

**Published:** 2026-09-11

**Authors:** Keisuke Tsuchida, Masahiko Okamoto, Akira Matsunobu, Takashi Kaneko, Yusuke Demizu, Tomoaki Okimoto, Masashi Koto, Yoshiyuki Shioyama, Hitoshi Ishikawa, Tatsuya Ohno, Hiroyuki Katoh, Reiko Imai, Japan Carbon-Ion Radiation Oncology Study Group

**Affiliations:** a Department of Radiation Oncology, Kanagawa Cancer Center, Kanagawa, Japan; b Department of Radiation Oncology, Gunma University Graduate School of Medicine, Gunma, Japan; c Department of Radiation Oncology, International Medical Center, Saitama Medical University, Saitama, Japan; d Ion Beam Therapy Center, SAGA-HIMAT Foundation, Saga, Japan; e Department of Radiation Oncology, Faculty of Medicine, Yamagata University, Yamagata, Japan; f Department of Radiology, Hyogo Ion Beam Medical Center, Hyogo, Japan; g QST Hospital, National Institutes for Quantum Science and Technology, Chiba, Japan

## Abstract

**Purpose:**

To evaluate the efficacy and safety of carbon-ion radiation therapy(C-ion RT) for patients with unresectable intermediate-category solitary fibrous tumor (SFT) in a nationwide multi-institutional observational study.

**Methods and Materials:**

This study included patients with unresectable intermediate-category SFT treated with C-ion RT at institutions affiliated with the Japan Carbon-Ion Radiation Oncology Study Group between April 2016 and December 2023. Primary endpoints were local control (LC) and overall survival. Secondary endpoints included progression-free survival, toxicity, and quality of life (QOL). Survival outcomes were analyzed using the Kaplan–Meier method. Toxicities were evaluated using the Common Terminology Criteria for Adverse Events version 5.0. QOL was assessed using the Functional Mobility Scale and EuroQol 5-Dimension 5-Level.

**Results:**

Ten patients from 3 institutions were enrolled in this study. The prescribed dose was 64.0 to 70.4 Gy in 16 fractions. The median follow-up was 49 months. Four-year overall survival, LC, and progression-free survival rates were 100% (95% CI, not available [NA]-NA), 100% (95% CI, NA-NA), and 67% (95% CI, 20%-90%), respectively. No local recurrences occurred, and 2 patients developed distant metastases. One patient experienced grade 2 late toxicity (peripheral neuropathy), and no grade ≥3 toxicities were observed. QOL scores were preserved throughout treatment. The median baseline EuroQol 5-Dimension 5-Level utility score was 0.85 (IQR, 0.76-0.97), and the median score at the final observation was 0.94 (IQR, 0.88-1.00), with no significant difference observed (*P* = .225).

**Conclusions:**

C-ion RT demonstrated favorable LC without severe toxicity, while maintaining QOL in patients with unresectable intermediate-category SFT.

## Introduction

Solitary fibrous tumor (SFT) is an ultrarare fibroblastic mesenchymal tumor characterized by a prominent staghorn vasculature and *NAB2-STAT6* gene fusion. SFTs can occur in any anatomic location, most often in pleural, meningeal, or extrapleural sites.^1^^,^^2^ The age-adjusted incidence rates are 0.61 and 0.37 per million persons per year for extra-meningeal and meningeal cases, respectively.^3^^,^^4^ A case series of extra-meningeal SFTs reported the following tumor localizations: limbs, 29%; intra-abdominal/pelvic, 30%; trunk/other, 19%; and pleural/intrathoracic, 22%.^5^ Extrathoracic SFTs have been reported to show a higher frequency of metastasis and a poorer prognosis than thoracic SFTs.^6^ The fifth edition of the World Health Organization classification of tumors of soft tissue and bone, subdivides SFTs into benign SFTs (intermediate category [locally aggressive]), SFT NOS (intermediate category [rarely metastasizing]), and malignant SFTs.^7^ Gold et al,^8^ in a single-institution retrospective study, reported that 20% of 79 SFTs were pathologically malignant. Even SFTs of the intermediate category without pathological features of malignancy may occasionally recur or metastasize. In recent years, prognostic risk models have been proposed to predict clinical outcomes more accurately than the conventional pathological classification into intermediate and malignant categories.^7^^,^^9^

The standard treatment for primary localized SFTs of the intermediate category is wide surgical resection. Perioperative radiation therapy (RT) using X-ray beams has been associated with better local control (LC) and overall survival (OS) than surgery alone.^10^^,^^11^ Haas et al^10^ reported that after propensity score matching in extra-meningeal SFTs, surgery plus RT was significantly associated with a lower risk of local progression. In a meta-analysis of intracranial SFTs, Na et al^11^ reported that surgery plus RT was associated with better progression-free survival (PFS) and OS than surgery alone. Alternatively, reports on treatment outcomes with definitive RT alone are limited, and only 1 was found, excluding those on stereotactic radiosurgery for intracranial SFTs. Haas et al^12^ reported that definitive RT without surgery resulted in 5-year LC and OS rates of 81.3% and 87.5%, respectively, in 16 patients with locally advanced or metastatic SFTs.

Carbon-ion RT (C-ion RT) has been employed for unresectable bone and soft tissue sarcomas (BSTS) in Japan since 1996. As C-ion RT has better dose distribution, it can deliver higher doses to the tumor than X-ray RT. C-ion beams have higher linear energy transfer than X-ray and proton beams, making them effective for treating radioresistant tumors, including BSTS. BSTS are radioresistant, necessitating high-dose irradiation for LC. C-ion RT has shown favorable outcomes in the treatment of unresectable BSTS.^13^, ^14^, ^15^ As the target of C-ion RT has been sarcomas, we have generally treated malignant SFTs thus far. Intermediate-category and malignant SFTs are locally aggressive, and depending on tumor location, surgery can sometimes be challenging. C-ion RT has been employed only for such complex intermediate-category SFT cases after tumor board review at each institute.^16^, ^17^, ^18^ Based on historical experience and after discussions with the Japan Carbon-Ion Radiation Oncology Study Group, including orthopedic surgeons specializing in sarcoma, a clinical trial was conducted to evaluate the feasibility of C-ion RT for intermediate-category SFTs.

This multi-institutional observational study aimed to evaluate the clinical outcomes of C-ion RT for intermediate-category SFTs, according to the World Health Organization fifth classification.

## Methods and Materials

### Study design

This multi-institutional observational study was conducted by the Japan Carbon-Ion Radiation Oncology Study Group, a nationwide collaborative clinical research group for C-ion RT in Japan. Clinical data were collected both prospectively and retrospectively. This study is registered with the University Hospital Medical Information Network Clinical Trials Registry (https://www.umin.ac.jp/ctr/index-j.htm), identification number UMIN000048444. The study included patients with intermediate-category SFTs treated with C-ion RT at participating institutions between April 2016 and December 2023. All patients provided written informed consent. The study protocol conformed with the principles of the Declaration of Helsinki and was approved by the institutional review board of each participating institution.

### Patient eligibility

Patients who initiated C-ion RT between April 2016 and December 2023 were enrolled. The study was conducted as a retrospective observational study from April 2016 until the date of institutional approval at each participating facility and as a prospective observational study thereafter. Patients who met all of the following criteria were eligible for inclusion: (1) histologically confirmed diagnosis of intermediate-category SFT; (2) considered not amenable to surgical resection; (3) Eastern Cooperative Oncology Group performance status 0 to 2; (4) age ≥10 years; (5) estimated life expectancy of ≥12 months; (6) written informed consent obtained from the patient; and (7) planned to receive C-ion RT in accordance with the standardized protocol of the Japanese Society for Radiation Oncology. Patients who met any of the following criteria were excluded: (1) diagnosis of malignant-category SFT; (2) presence of severe comorbidities precluding treatment tolerance; (3) concurrent administration of systemic therapies, including chemotherapy, molecular targeted therapy, or immunotherapy, during the course of irradiation; (4) active and treatment-resistant infection within the planned irradiation field; or (5) considered unsuitable for participation by the attending physician based on clinical, psychological, or other factors.

Pathologic diagnoses were locally reviewed by reference sarcoma pathologists at each institute. In cases of pathologic diagnostic uncertainty, other sarcoma-specialized pathologists were consulted.

### Treatment

**Figure 1:**
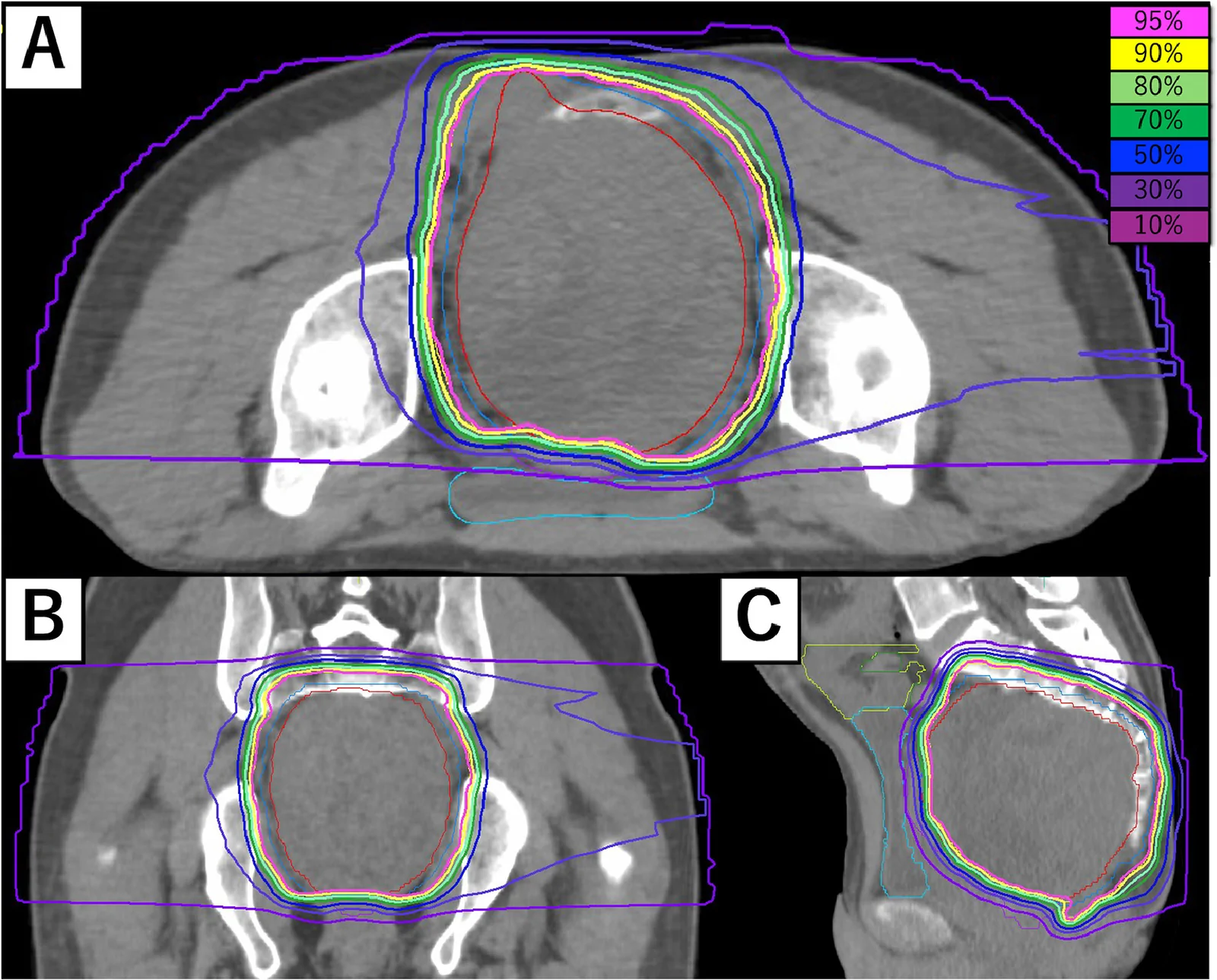
A 38-year-old male patient with a solitary fibrous tumor in the presacral region treated with carbon-ion radiation therapy (70.4 Gy in 16 fractions). Dose distribution on (A) axial, (B) coronal, and (C) sagittal computed tomography images. The area outlined in red indicates the gross tumor volume. The colored areas represent the 95% (pink), 90% (yellow), 80% (light green), 70% (green), 50% (blue), 30% (purple), and 10% (light purple) isodose curves.

A representative C-ion RT protocol is described below. Treatment planning was conducted using a computed tomography (CT)-based 3-dimensional treatment planning system. Patients were immobilized using custom-made thermoplastic shells and body rests. Depending on tumor location, patients were positioned for treatment in the supine, prone, or both positions. The gross tumor volume and organs at risk were delineated on the planning CT images with reference to magnetic resonance imaging and positron emission tomography–CT images as appropriate. The clinical target volume (CTV) was defined as the gross tumor volume plus a margin of at least 5 to 10 mm, which was adjusted as appropriate based on the pattern of tumor extension and the anatomical relationship with surrounding organs. The planning target volume was delineated by adding a 5 mm margin to the CTV, with modifications made as necessary considering the proximity of adjacent organs and setup reproducibility in the passive scanning setting. In the active scanning setting, the planning target volume was determined based on the tumor shape and location, with margins ranging from 1 to 5 mm beyond the CTV. Dose fractionation was prescribed according to the standardized treatment policy of a national radiation oncology society, and a total dose of 64.0 to 70.4 Gy in 16 fractions was delivered (relative biological effectiveness weighted dose based on the modified microdosimetric kinetic model).^19^^,^^20^ In accordance with other BSTS protocols, 70.4 Gy was generally selected. However, for tumors located in the head and neck, skull base, or paraspinal regions, the dose was reduced to 64.0 Gy to limit the risk of severe toxicity to adjacent critical organs, particularly the spinal cord and brainstem. The major dose constraints for organs at risk used at the institution with the largest number of patients in this study are summarized in Table E1. Irradiation was performed 4 times per week over a treatment period of 4 weeks. C-ion beams were administered via passive scattering or scanning techniques, depending on the treatment system used at each participating institution. A representative dose distribution is shown in Fig. 1.

### Data collection and statistical analysis

Given the observational nature of the study, follow-up procedures were performed according to the institutional policies of each participating center. Clinical data and radiotherapy details were obtained from institutional databases, patient medical records, and treatment planning systems at each participating center. The follow-up period was calculated from the date of C-ion RT initiation. The primary endpoints were LC and OS rates. The secondary endpoints included assessments of physical function, incidence of distant metastasis, PFS, and incidence of late toxicity (graded according to Common Terminology Criteria for Adverse Events version 5.0). Exploratory endpoints included quality of life (QOL).

OS, LC, and PFS curves were estimated using the Kaplan–Meier method. Continuous variables are presented as medians and IQRs, and categorical variables are presented as frequencies and percentages. Tumor response was evaluated according to the Response Evaluation Criteria in Solid Tumors version 1.1, using the maximum tumor diameter at baseline before treatment and at the time of the final imaging assessment. Functional mobility was assessed using the Functional Mobility Scale (FMS).^21^ Health-related QOL was assessed using the EuroQol 5-Dimension 5-Level (EQ-5D-5L) questionnaire on visits to the hospital.^22^ The FMS was selected because it enables the assessment of patients’ ambulatory function, whereas the EQ-5D-5L was selected because it enables a comprehensive evaluation of patients’ health status. In addition, information on both measures was retrospectively available from the participating institutions, which was also a factor in the selection of these measures for this study. EQ-5D-5L utility scores were calculated using the Japanese value set for the EQ-5D-5L.^23^ Differences in EQ-5D-5L utility scores between baseline and final observation were assessed using the Wilcoxon signed-rank test. A *P* value ≤.05 was considered statistically significant. All statistical analyses were performed using R software (version 4.5.0; R Foundation for Statistical Computing).

## Results

### Patient and treatment characteristics

The number of patients enrolled from Kanagawa Cancer Center, Gunma University Heavy Ion Medical Center, and QST Hospital was 5, 4, and 1, respectively. Four patients were observed retrospectively, 1 prospectively, and 5 during both the retrospective and prospective observation periods. Patient and treatment characteristics are summarized in Table 1. The median age was 56 years (IQR, 51-67 years), and the cohort included 4 men and 6 women. Six patients had newly diagnosed tumors, 3 had postoperative recurrent tumors, and 1 had a metastatic tumor. The primary and irradiated tumor sites varied across multiple anatomical locations. The median tumor size was 71 mm (IQR, 50-86). Scanning beam delivery was used in 6 patients, and passive beam delivery (layer-stacking irradiation) was used in 4 patients. A total dose of 70.4 Gy was delivered in 7 patients and 64.0 Gy in 3 patients, all administered in 16 fractions.

**Table 1:** Patient and treatment characteristics

Characteristics	N = 10
Age, y	56 (51-67)
Follow-up period, mo	49 (30-51)
Sex	
Male	4
Female	6
Performance status	
0	4
1	5
2	1
Tumor type	
Primary tumor with no prior treatment	6
Recurrent tumor after resection	3
Metastatic tumor	1
Reason for C-ion RT	
Unresectable	9
Patient’s refusal of surgery	1
Primary site	
Intracranial region	1
Skull base	1
Posterior cervical region	1
Vertebra	2
Retroperitoneum	1
Presacral region	3
Pelvic floor	1
Irradiated site	
Skull base	1
Posterior cervical region	1
Vertebra	2
Retroperitoneum	1
Presacral region	3
Pelvic floor	1
Ischium	1
Maximum diameter of tumor, mm	71 (50-86)
Spacer placement	
Yes	0
No	10
Total irradiation dose (Gy in 16 fractions)	
70.4	7
64.0	3
Irradiation method	
Scanning	6
Passive (Layer-stacking)	4

*Abbreviations:* C-ion RT = carbon-ion radiation therapy; RT = radiation therapy.

Note: data are presented as median (interquartile range) or number of patients.

### Efficacy and toxicity

**Figure 2:**
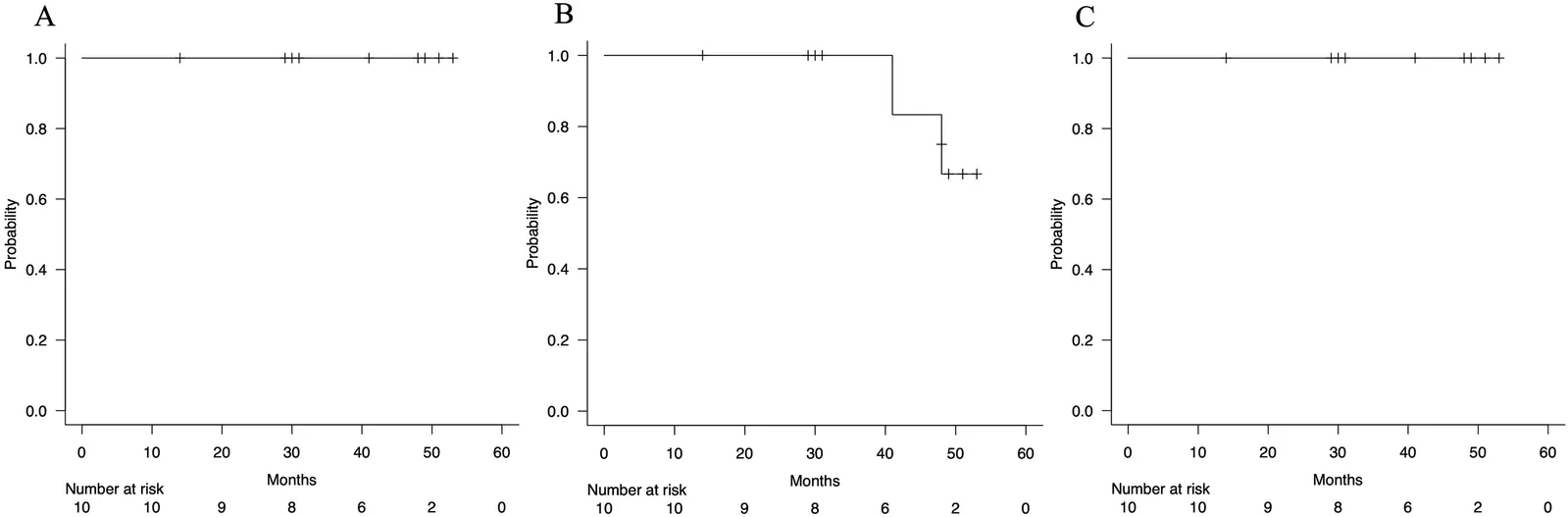
Kaplan–Meier curves for overall survival (A), progression-free survival (B), and local control (C) in all patients.

The median follow-up was 49 months (IQR, 30-51). Among the 10 patients, 4-year OS, PFS, and LC rates were 100% (95% CI, not available [NA]-NA), 67% (95% CI, 20%-90%), and 100% (95% CI, NA-NA), respectively (Fig. 2). Two patients had distant metastases: 1 to the rib and 1 to the femoral bone. Detailed clinical data for all patients are presented in Table 2. At the time of the final imaging assessment, the median percentage change in tumor size was −32% (IQR, −43% to −16%) compared with baseline before C-ion RT. According to the Response Evaluation Criteria in Solid Tumors version 1.1 criteria, 1 patient achieved a complete response, 4 showed partial responses, and 5 had stable disease.

**Table 2:** Detailed clinical characteristics of all patients.

Patient (sex)	Age	Primary site	Irradiated site	Tumor type	Diameter (mm)	RT dose (Gy)	Follow-up (mo)	Status at last follow-up	Change in tumor size* (RECIST)	Type of recurrence (Recurrent site)	Time to recurrence	≥G2 late toxicity
1 (M)	38	Presacral region	Presacral region	Treatment naïve	98	70.4	48	NER	−61% (PR)	No	NA	No
2 (F)	56	Cervical vertebra	Cervical vertebra	Local recurrence after surgery	17	70.4	49	NER	−100% (CR)	No	NA	No
3 (M)	51	Intracranial region	Ischium	Metastasis after surgery	75	70.4	49	AWD	0% (SD)	Distant metastasis (Rib)	41 mo	No
4 (F)	69	Skull base	Skull base	Local recurrence after surgery	46	64.0	53	NER	−13% (SD)	No	NA	No
5 (F)	51	Pelvic floor	Pelvic floor	Treatment naïve	97	70.4	51	NER	−46% (PR)	No	NA	No
6 (M)	40	Presacral region	Presacral region	Treatment naïve	88	70.4	54	AWD	−35% (PR)	Distant metastasis (Femur)	48 mo	G2 PN
7 (F)	67	Presacral region	Presacral region	Treatment naïve	60	70.4	31	NER	−35% (PR)	No	NA	No
8 (F)	74	Thoracic vertebra	Thoracic vertebra	Local recurrence after surgery	32	64.0	30	NER	−25% (SD)	No	NA	No
9 (M)	65	Posterior neck	Posterior neck	Treatment naïve	67	64.0	29	NER	−28% (SD)	No	NA	No
10 (F)	56	Retroperitoneum	Retroperitoneum	Treatment naïve	81	70.4	14	NER	−12% (SD)	No	NA	No

*Abbreviations:* AWD = alive with disease; F = female; M = male; NER = alive with no evidence of recurrence; PN = peripheral neuropathy; RECIST = Response Evaluation Criteria in Solid Tumors; TRR = Tumor reduction ratio.

⁎ The size change represents the change in the longest tumor diameter from baseline (before treatment initiation) to the final imaging assessment.

No cases of acute toxicity of grade ≥2 were observed. One patient experienced grade 2 peripheral neuropathy (pain) as a late toxicity 12 months after treatment initiation, which improved to grade 1 by 20 months.

### QOL

Before C-ion RT, the FMS scores were 6 in 7 patients and 4 in 3 patients, with all patients maintaining the same scores at the final follow-up, which ranged from 1 to 53 months after C-ion RT. One patient had pretreatment urinary dysfunction, 3 had other neurological impairments, and 2 experienced pain; none of these symptoms showed any change at the final observation. No fractures occurred during the follow-up period.

**Figure 3:**
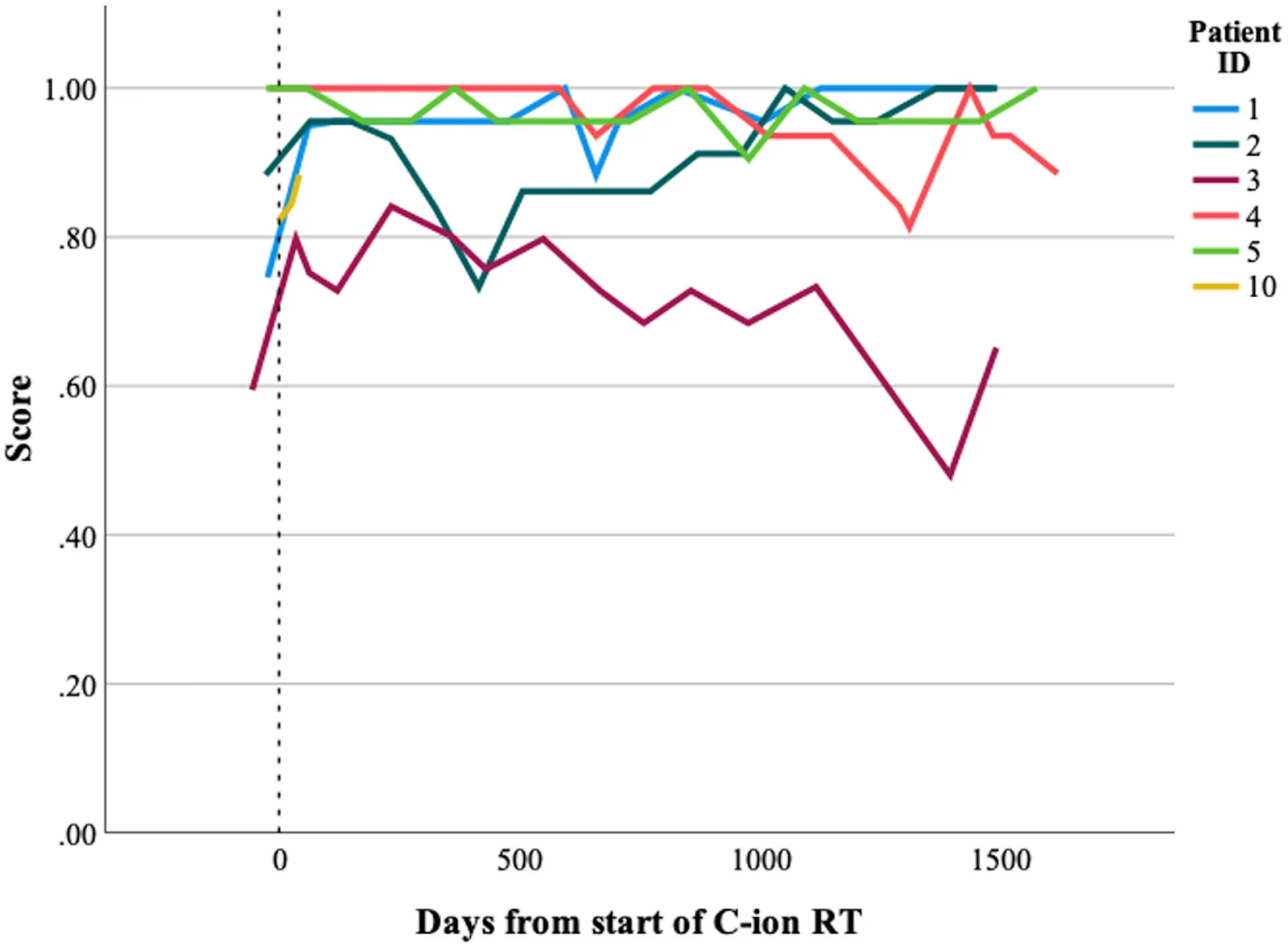
Longitudinal changes in EuroQol 5-Dimension 5-Level utility scores in each patient. The patient IDs correspond to those in Table 2.

EQ-5D-5L data were obtained from 6 patients. The 4 patients who were followed retrospectively only and for whom EQ-5D-5L data had not been collected prior to the initiation of this study were treated as missing. The longitudinal changes in EQ-5D-5L scores for each patient are shown in Fig. 3. The median pretreatment baseline score was 0.85 (IQR, 0.76-0.97), and the median score at the final observation was 0.94 (IQR, 0.88-1.00), with no significant difference observed (*P* = .225).

## Discussion

This nationwide multi-institutional observational study reports the treatment outcomes of C-ion RT for unresectable intermediate-category SFTs. To date, reports on RT for unresectable SFTs in a curative setting remain scarce. In this study, C-ion RT demonstrated favorable LC, a low incidence of toxicities, and preservation of FMS and QOL, suggesting that it could be a promising treatment option for SFTs.

In X-ray RT for unresectable SFTs, Haas et al^12^ reported that 16 patients (including 2 with malignant SFTs) treated with definitive RT, typically 60 Gy delivered in 30 fractions, achieved 2- and 5-year LC rates of 93.8% and 81.3%, respectively. In the same study, 24 patients (including 9 with malignant SFTs) who underwent palliative RT, predominantly 39 Gy delivered in 13 fractions, had 2- and 5-year LC rates of 70.8% and 62.5%, respectively. For intracranial SFTs, which were previously referred to as hemangiopericytomas, stereotactic irradiation using modalities such as gamma knife radiosurgery has been reported. Cohen-Inbar et al^24^ reported that 90 patients with cranial SFT (21% of whom had malignant SFT) underwent stereotactic radiosurgery using gamma knife, with 2- and 4-year LC rates of 81.7% and 66.3%, respectively. Copeland et al^25^ reported clinical outcomes of stereotactic radiosurgery in 22 patients with meningeal SFT, with 3- and 5-year LC rates of 68% and 59%, respectively. C-ion RT in the present study achieved a 4-year LC rate of 100%. Although this outcome may appear favorable compared with the 5-year LC rate of 81.3% reported for definitive X-ray RT by Haas et al, such comparisons are inherently limited by the small sample size of the present cohort and the lack of a direct comparative study design. According to the report by Haas et al,^12^ LC was more favorable with definitive radiation doses than with palliative doses, suggesting that LC with X-ray RT is dose-dependent. In C-ion RT, the strong LC observed in this study may be attributed to the high-dose delivery enabled by the superior dose conformity of C-ion RT, as well as its inherently higher biological effectiveness, similar to other malignant BSTS. Alternatively, due to the lack of an adequate direct contemporary control group treated with X-ray or proton beam therapy, it remains unclear whether the observed outcomes are attributable to the antitumor effect of C-ion RT itself or to patient selection. In the present study, a total dose of 70.4 Gy was used as the standard; however, considering the risk of toxicities, a reduced dose of 64.0 Gy was selected depending on the tumor site. LC was achieved with both dose levels. Nevertheless, since long-term LC remains unclear and the observed toxicities were minimal, we consider 70.4 Gy to be generally recommended at present, while dose reduction may be an acceptable option depending on the anatomical site. In addition, the report by Haas et al^12^ indicated a tendency for LC rates with X-ray RT to decline in the very long term, beyond 5 years. Assessment of long-term LC beyond 5 years is considered an important issue to be addressed in future studies of C-ion RT, particularly to determine whether its theoretically higher antitumor efficacy can translate into sustained ultra–long-term disease control.

In the present study, distant metastatic recurrence was observed in 2 patients (20%). Intermediate-category SFTs are clinically heterogeneous, making their clinical course difficult to predict.^26^ Although the majority of tumors follow a benign course, 5% to 10% of cases develop recurrence or metastasis, typically to the lungs, liver, or bones.^26^ Notably, metastases may occasionally occur even in tumors that exhibit histologically benign features.^26^ Demicco et al^9^ proposed a risk stratification model for predicting metastatic potential based on 4 factors: age, tumor size, mitotic count, and tumor necrosis.^7^ More recently, risk models incorporating additional biomarkers have also been proposed.^2^ Although such risk classification was not applied in the present study, follow-up strategies and treatment planning based on risk stratification may be important in the future. Chemotherapy for SFT has not yet been standardized. Broto et al. conducted a phase II study of pazopanib in patients with locally advanced or metastatic typical SFT, corresponding to the current intermediate category. They reported that pazopanib showed better results in this typical SFT cohort than in previous controls treated with chemotherapy, in terms of both PFS and OS.^27^ Evidence for (neo)adjuvant chemotherapy in patients with localized, resectable SFT is limited.^28^ However, in those at high risk of recurrence, adding systemic therapy to local treatment may improve clinical outcomes.

In the present study, only 1 patient (10%) who received irradiation to the presacral region experienced grade ≥2 toxicity, specifically grade 2 late peripheral neuropathy (pain). Generally, the risk of toxicities associated with C-ion RT is dependent on the irradiated site and irradiated doses. In a multi-institutional observational study of C-ion RT for sacral chordoma, Demizu et al. reported a 6% incidence of grade ≥3 late toxicities, with the majority of grade 4 events being skin-related.^29^ Imai et al^30^ reported a 3% incidence of grade ≥3 late toxicities in patients with axial soft tissue sarcomas treated with C-ion RT. Jingu et al^31^ reported a 19% incidence of grade ≥3 late toxicities in patients with head and neck sarcomas treated with C-ion RT. In the present study, despite including tumors primarily in the pelvic region as well as some in the skull base and neck, treatment was completed without severe toxicity. This favorable outcome is partly attributable to the use of advanced irradiation techniques, such as layer-stacking and scanning methods, which enable more precise dose delivery while sparing surrounding organs.^32^

In this study, FMS and EQ-5D-5L were used to assess QOL. Among patients with available data, no deterioration in QOL was observed after C-ion RT. Komatsu et al. reported on QOL outcomes following C-ion RT for BSTS, using the Musculoskeletal Tumor Society scoring system and the physical and mental component scores of the Short Form-8. They found that both the Musculoskeletal Tumor Society and the physical component score of Short Form-8 were preserved, whereas the mental component score improved.^33^ The present findings are consistent with this previous report, indicating that C-ion RT preserves QOL. These results suggest that C-ion RT is a well-tolerated local treatment modality with minimal impact on QOL.

This study has several limitations. First, the sample size was small, with only 10 patients enrolled, limiting the statistical power and generalizability of the findings. Second, although this was a multi-institutional observational study, the majority of cases were treated retrospectively. This design introduces potential selection bias and inconsistency in treatment and follow-up protocols for each institution. In addition, regarding QOL data, EQ-5D-5L data were available for only 6 of the 10 patients, indicating a potential risk of bias. Third, the median follow-up period, although relatively long, may still be insufficient to fully assess long-term outcomes, including late toxicity and very late recurrences, which are known to occur in patients with SFT. Therefore, further investigation is required to determine whether C-ion RT can achieve sustained ultra–long-term LC. Nevertheless, given the extreme rarity of unresectable intermediate-category SFT, this nationwide report on the clinical outcomes of C-ion RT is valuable and provides important insights for future therapeutic development.

## Conclusions

C-ion RT demonstrated favorable LC without severe toxicity, while maintaining QOL in patients with unresectable intermediate-category SFT. These preliminary findings suggest that C-ion RT may be a promising treatment option. However, given the lack of a comparative cohort, small sample size, and limited follow-up duration, these findings should be interpreted with caution. Further studies with larger patient cohorts and sufficiently long-term follow-up to capture very late events are required to confirm its efficacy and safety.

## Disclosures

Hiroyuki Katoh reports financial support was provided by Toshiba Energy Systems and Solutions Corporation. If there are other authors, they declare that they have no known competing financial interests or personal relationships that could have appeared to influence the work reported in this paper.

## Appendix Supplementary materials

Supplementary materials Image, application 1
