# Supplementary material for: Carbon-Ion Radiation Therapy for Unresectable Solitary Fibrous Tumor of the Intermediate Category: A Nationwide Multi-institutional Observational Study by the Japan Carbon-Ion Radiation Oncology Study Group
Source: Adv Radiat Oncol. 2026 Sep 11;11(9):102143. doi: 10.1016/j.adro.2026.102143 (PMC13576702; doi:10.1016/j.adro.2026.102143)
Supplement: Supplementary file 1 [file mmc1.pdf]

Table E1. Major dose constraints used in the present study (16 fractions)

Organ	Parameter	Constraint
Spinal cord	Dmax	30 Gy*
Brainstem	Dmax	30 Gy
Skin	$D0.5cm^3$	54–60 Gy
Gastrointestinal tract	$D1–2cm^3$	44–46 Gy
Bladder	$D2cm^3$	60 Gy

Abbreviations: Dmax, maximum point dose within the organ at risk; $Dxxcm^3$, minimum dose to the most irradiated contiguous xx cm^3 of the organ at risk.

* Gy represents the relative biological effectiveness-weighted dose based on the modified microdosimetric kinetic model.
